# Female and male life tables for seven wild primate species

**DOI:** 10.1038/sdata.2016.6

**Published:** 2016-03-01

**Authors:** Anne M. Bronikowski, Marina Cords, Susan C. Alberts, Jeanne Altmann, Diane K. Brockman, Linda M. Fedigan, Anne Pusey, Tara Stoinski, Karen B. Strier, William F. Morris

**Affiliations:** 1 Department of Ecology, Evolution and Organismal Biology, Iowa State University, Ames, Iowa 50011, USA; 2 Department of Ecology, Evolution and Environmental Biology, Columbia University, New York, New York 10027, USA; 3 Department of Biology, Duke University, Durham, North Carolina 27708, USA; 4 Institute of Primate Research, National Museums of Kenya, Nairobi, Kenya; 5 Department of Ecology and Evolutionary Biology, Princeton University, Princeton, New Jersey 88001, USA; 6 Department of Anthropology, University of North Carolina, Charlotte, North Carolina 28223, USA; 7 Department of Anthropology, University of Calgary, Calgary, Alberta, Canada T2N 1N4; 8 Department of Evolutionary Anthropology, Duke University, Durham, North Carolina 27708, USA; 9 The Dian Fossey Gorilla Fund International and Zoo Atlanta, Atlanta, Georgia 30315, USA; 10 Department of Anthropology, University of Wisconsin-Madison, Madison, Wisconsin 53706, USA; 11 These authors contributed equally to this work

**Keywords:** Zoology, Ageing, Evolution, Evolutionary ecology

## Abstract

We provide male and female census count data, age-specific survivorship, and female age-specific fertility estimates for populations of seven wild primates that have been continuously monitored for at least 29 years: sifaka (*Propithecus verreauxi*) in Madagascar; muriqui (*Brachyteles hypoxanthus*) in Brazil; capuchin (*Cebus capucinus*) in Costa Rica; baboon (*Papio cynocephalus*) and blue monkey (*Cercopithecus mitis*) in Kenya; chimpanzee (*Pan troglodytes*) in Tanzania; and gorilla (*Gorilla beringei*) in Rwanda. Using one-year age-class intervals, we computed point estimates of age-specific survival for both sexes. In all species, our survival estimates for the dispersing sex are affected by heavy censoring. We also calculated reproductive value, life expectancy, and mortality hazards for females. We used bootstrapping to place confidence intervals on life-table summary metrics (*R*_0_, the net reproductive rate; *λ*, the population growth rate; and *G*, the generation time). These data have high potential for reuse; they derive from continuous population monitoring of long-lived organisms and will be invaluable for addressing questions about comparative demography, primate conservation and human evolution.

## Background & Summary

The life table has a long history as a means of summarizing census and birth data to describe how survival and fertility vary with age, and of using these underlying parameters to estimate useful population-level statistics^1^. Specifically, age-specific survivorship *l*_*x*_ and fertility *m*_*x*_, (where *x* indicates a discrete age class) are all that are needed to estimate the relative contribution of an age *x* individual to the future size of the population (the ‘reproductive value’, *v*_*x*_), the life expectancy of an age *x* individual (*e*_*x*_), the average lifetime reproductive success (the average lifetime number of daughters per mother, *R*_*0*_), the asymptotic population growth rate (*λ*), the stable fraction of individuals of age *x* in a steadily growing or declining population (the age *x* entry in the ‘stable age distribution’, *w*_*x*_), and the average number of years between a female’s birth and the birth of her offspring (the ‘generation time’, *G*).

Generating high-quality life tables for long-lived species living in the wild is challenging for various reasons, with the long-term commitment to individual-based monitoring foremost among them. Given that the life table is the most fundamental tool in population biology for inferring the dynamics of age-structured populations, we sought to combine, analyse, and make available the life table data from our long-term studies of globally distributed primate populations^2^. We have used these data to report on aging rates^3^, reproductive senescence rates^4^ and population sensitivity to environmental variation^5^. Here, we present sex-specific raw data in the form of census counts binned by age, as well as estimated survival and female fertility rates with details on their calculations. We then estimate important population parameters (age specific reproductive values, life expectancies, net reproductive rates, and generation time) from the female life tables to demonstrate their utility and variation among the taxa represented in our sample.

Primate survival and birth rates are of primary importance in the literature that addresses variation in human demographic patterns (e.g., (ref. 6) and chapters therein). Our study includes representatives from all major lineages of the order Primates: one strepsirrhine, two New World monkeys, two Old World monkeys and two great apes. Long-term individual-based study of a focal population of each of these species began between 1963 and 1984. All studies are continuous since their inception, are ongoing at present, and involve year-round monitoring of individuals. Monitoring occurs regularly, but varies from near-daily to monthly censuses, in some cases with seasonal variation and/or irregular gaps, especially for the non-philopatric sex. This long-term monitoring allows for nearly complete survival and fertility records for females and survival records for males (excepting male blue monkeys; see below, with the caveat that survival estimates for the dispersing sex in each species are affected by frequent censoring. Table 1 includes information on the overall characteristics of each study population, including the predominant dispersing sex and median age at first dispersal as well as sample sizes for males and females.

The purpose of this data descriptor was two-fold. First, our goal was to create, analyse, and make available high-quality life tables for females, and survivorship tables for males, at all ages. Individuals were monitored from first contact (i.e., either birth or first observation) through last contact (i.e., death, permanent disappearance, or end of observation while still alive, the latter of which is a right censored observation). From these individual-based regular census observations, we reduced the complexity of the data by generating *synthetic* (i.e., collapsing across all study years) life-tables^7^ and binning individuals into one-year age intervals^3^. Second, we estimated age-specific female fertility. These calculations included observed live births for known-age females, and were adjusted for number of females within the age-class and for possible missed pregnancies during gaps in fertility monitoring. Because our life tables are synthetic, we do not account for changes in mortality and fertility due to year-to-year environmental variation^5^ or to changes in population size over the course of the study^8,9^.

## Methods

To estimate survivorship and mortality rates, we constructed sex-specific life-tables including age-specific vectors of entries, deaths, permanent disappearances, and censored individuals from the census data (Table 1). Counts of these entries, deaths, permanent disappearances, and censors for each 1-year age-class can be found in Tables 2A and 2B in Data Citation 1 for females and males, respectively. (Data for male blue monkeys are truncated at age 7 due to unknown ages for immigrant adult males and mostly unknown fates for emigrant males.) (Data Citation 1 is available from the Dryad Digital Repository: http://dx.doi.org/10.5061/dryad.v28t5).

For most individuals in each study population, individual ages were known to within a fraction of a year. Exceptions occurred for individuals that were present at the start of each study or that immigrated into the study population. We estimated ages for these animals based on physical characteristics and known aging patterns in these populations; details of age estimation procedures are described in ref. 2. See the section ‘Technical Validation’ below for how we assessed the impact of this uncertainty.

For each 1-year age interval *x*, individuals either survive the age interval and transition into the subsequent interval; enter the population (*t*_*x*_, where *t*_*0*_ represents births); die (*d*_*x*_); are right-censored (*c*_*x*_, defined in this data descriptor as either a known successful emigration or end of observation); or permanently disappear (*p*_*x*_). For this report, we add permanent disappearances (*p*_*x*_) to the deaths so as not to underestimate mortality (although in some cases this likely overestimates mortality). This addition of *p*_*x*_ to *d*_*x*_ differs from our earlier work^3^ in which we treated these animals as right-censored. Henceforth, we refer to this sum of the original *d*_*x*_ and *p*_*x*_ as *dp*_*x*_.

To estimate mortality at age *x*, *q*_*x*_, we used the actuarial method, which subtracts half of the censored individuals from the number alive at age *x* (assuming that half of the censoring occurred before and half after the midpoint of the age interval) to obtain a censoring-adjusted sample size (*N*_*q.x*_) that was used to compute *q*_*x*_ as *dp*_*x*_*/N*_*q.x*_. From the actuarial estimates of the *q*_*x*_ values, we computed the survivorship (*l*_*x*_) values from

(1)l0=1andlx=∏i=0x−1(1−qi);x>0.

For species in which the oldest individuals are still alive (i.e., are right-censored), the last estimate for age-specific mortality and survivorship occurs in the last age interval with an observed death.

**Figure 1:**
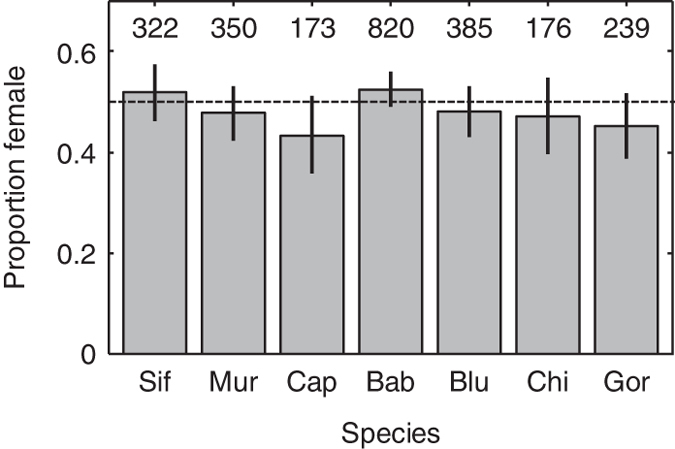
Sex ratio (proportion female) for individuals born into each study population whose sex was determined before they died. The total number of sexed offspring is shown at the top of each bar. Error bars show the 95% binomial confidence intervals for the proportion female, and overlap 0.5 (dashed line) for all species. We thus justify the 50:50 sex ratio we assumed when estimating fertilities.

**Figure 2:**
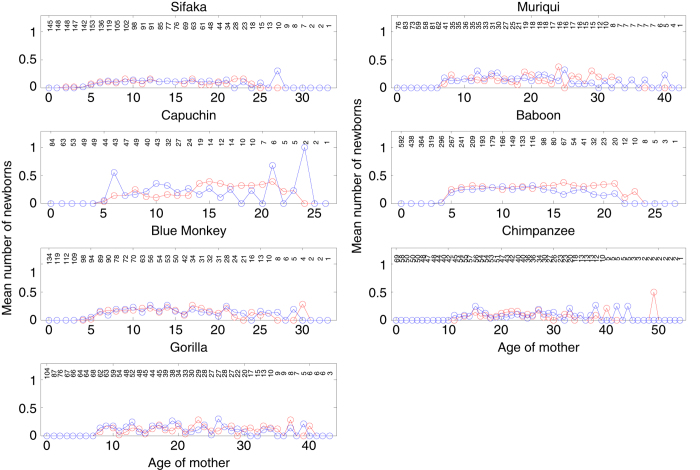
Age-specific probability of producing a son (blue) versus a daughter (red). Plotted values are the weighted number of sons or daughters divided by the weighted number of mothers (*N*_*m.x*_ in Table 2A in Data Citation 1). Numbers at the top of each panel give the (unweighted) number of mothers in each age interval. There is no striking evidence that offspring sex ratio varies with the age of the mother. These estimates necessarily ignore offspring that died before their sex could be determined, which are not ignored in the estimates of *m.x* in Table 2A in Data Citation 1.

In computing age-specific fertilities (*m*_*x*_ values), which in our studies were the expected number of daughters born to females, we accounted for the fact that not all females were observed intensively during all age intervals, such that some live births may have been missed. Specifically, we computed for each female the fraction of a given age interval during which she was under intensive observation, and used that fraction as a weighting factor: we multiplied the observed number of live births in the interval for each female by her weighting factor, summed these products over all females observed at that age, and divided this sum by the sum of the weighting factors across all those females. Because sample sizes for estimating fertilities (*N*_*m.x*_) were small for some species, we reduced the influence that demographic stochasticity would have had on our *m*_*x*_ values had we included only daughters in the number of live births by instead including all newborns and then dividing the resulting total fertilities by 2 to get the average number of daughters per female of age *x*. This procedure also allowed us to include newborns that died before their sex could be determined, which represented a substantial fraction of newborns for some of the study species. The assumption of a 50:50 sex ratio at birth is justified for all species in our study (Fig. 1), and there is also no evidence that the offspring sex ratio varies with the age of the mother (Fig. 2).

We computed *R*_*0*_, the net reproductive rate (lifetime number of daughters per female) and *G*, the generation time (number of years between a mother’s birth and the birth of her average daughter), directly from the *l*_*x*_ and *m*_*x*_ values as

$$R_{0}=\sum_{x=0}^{\omega }l_{x}m_{x}\;\;\mathrm{and}\;G=\left(\sum_{x=0}^{\omega }xl_{x}m_{x}\right)/R_{0}$$

where *ω* is the age at which *l*_*x*_ either reaches zero or is truncated after the last observed death. From the Leslie matrix version of the life table, we computed the asymptotic population growth rate (dominant eigenvalue), stable age distribution (dominant right eigenvector), and the vector of reproductive values (dominant left eigenvector)^10^. We computed the life expectancy at each age *x* by summing each column of the fundamental matrix (the inverse of the difference between the identity matrix and the Leslie matrix with the fertility terms replaced by zeros^11^).

To place confidence limits on the life history statistics, we used a parametric bootstrap to sample from the distributions of the *l*_*x*_ and *m*_*x*_ values. To produce a sample of *l*_*x*_ values, we used the actuarial estimate of each *q*_*x*_ value and the number of individuals at risk of dying at that age to draw a binomial number of individuals dying which, divided by the number at risk, yielded one plausible *q*_*x*_ value. Because the number at risk under the actuarial method may include fractions, we first rounded up the number at risk to the next highest integer. From each set of *q*_*x*_ values, we computed the *l*_*x*_ values using equation (1). We sampled each *m*_*x*_ value by drawing a number of daughters for a female from a Poisson distribution with mean equal to the best estimate of the *m*_*x*_ value, repeating this random draw for the number of females whose fertility was observed at age x, summing the total number of offspring produced, dividing this sum by the number of females to yield one plausible *m*_*x*_ value, and repeating for all ages. Use of the Poisson distribution for the number of offspring accounts for the fact that, while most females produce only 0 or 1 offspring in an age interval, successive births for a female sometimes fall within the same age interval—such as in the case of an infant death—and there is occasional but rare twinning in our study species. Because the weighting procedure to account for possible missed births that we described above yielded a sum of weights that was not an integer (see Table 2A in Data Citation 1), we rounded this sum up to the next highest integer and used the result for the number of females at age *x*. From the sampled set of *l*_*x*_ and *m*_*x*_ values, we computed all the life history statistics reported in the text and stored them. We repeated this procedure 4999 times and then identified the 2.5th and 97.5th percentiles of the bootstrap distribution for each statistic as the lower and upper confidence limits, respectively. Custom-built code to perform the parametric bootstrap was written in R^12^.

## Data Records

The data are organized into tables and have been deposited in the Dryad Digital Repository (Data Citation 1: http://dx.doi.org/10.5061/dryad.v28t5). Table 1 (above) provides an overview of the species, locations, and sample sizes. Table 2A of Data Citation 1—‘Female Life Tables’—organizes the counts of female deaths, disappearances, censors, and entries, as well as sample sizes for fertilities and mortality, all by one-year age intervals. In addition to these counts, Table 2A of Data Citation 1 includes estimates for age-specific mortality, fertility, survivorship, reproductive value, life expectancies, and stable age distribution (with upper and lower 95% confidence limits, as described above). Table 2B of Data Citation 1—‘Male Life Tables’—organizes the counts of male deaths, disappearances, censors, and entries, as well as sample sizes for mortality, in one-year age intervals. In addition to the counts, Table 2B of Data Citation 1 includes estimates of age-specific mortality and survivorship, with upper and lower 95% confidence limits. Table 3 of Data Citation 1 contains population parameters computed from the female life tables, namely net reproductive rate, generation time, fraction of newborns surviving to the median age at which females first reproduced, and population growth rate. Table 4 of Data Citation 1 contains an example of a life table converted to a Leslie Matrix for female baboons (see Usage Notes below).

## Technical Validation

Two challenges in data collection are relevant to the reuse of these data. First, when field studies began, ages of animals were largely unknown. Second, the determination of the fate of individuals is usually indirect. Here we discuss both of these issues and the measures that we took to address them.

### Age estimates

In each population, some individuals were present as immatures or adults when observations commenced, and some individuals of unknown ages migrated into the study population. For these animals, whose ages were estimated rather than known, we assigned a minimum and maximum value for their age (see ref. 2 for details). For most animals, the range of possible ages did not exceed a 1-year interval. However, for some individuals, particularly those that were first observed as adults at the onset of a study, age estimates had large ranges.

To test how this uncertainty in age affected our estimates of mortality, we conducted sensitivity analyses assigning individuals to their oldest possible age and their youngest possible age and tested for an effect on estimates of initial adult mortality and rate of aging^3^. We found that because the uncertainty was distributed across the adult lifespan, and no single age interval was dominated by individuals of uncertain age, the effects on mortality estimates were negligible. For the same reason, we expect the effect of age uncertainty on age-specific fertility to be negligible.

### Animals that disappeared with unknown fate

In many cases of animal disappearance it was possible to assign the fate. For example, infant males and females are unable to survive without maternal care and so infant-only disappearances were certain deaths. For adult animals, the degree of uncertainty of fate varied between the sexes. In species characterized by sex-biased dispersal, members of the non-dispersing sex that disappear can reliably be assigned to deaths. For each study included in this data descriptor, long-term monitoring (at least 29 years) has verified that members of the non-dispersing sex that disappear rarely move between groups and do not reappear; death was assigned in all such cases. For the dispersing sex, by contrast, it is often impossible to distinguish between disappearances caused by death versus disappearances caused by departures from the study population. In all such cases, we used expert knowledge to judge whether a death had occurred. For example, if an animal was wounded, showed difficulty foraging or keeping up with the group, and then went missing, we classified it as a death. However, in many cases, animals (particularly of the dispersing sex) disappeared with no evidence of morbidity. In Tables 2A and 2B (Data Citation 1), we distinguish the disappearances that were assumed to represent deaths (*d_x_*) from those for which we were unable to differentiate between death and emigration outside of the study population to a satisfactory degree based on contextual information (*p_x_*). The sole exception is for male blue monkeys, which are truncated at age 7 because the majority of emigrating males have unknown fate. We recommend cautious interpretation of mortality estimates that treat disappearances in the *p*_*x*_ column either as deaths or as censored individuals; doing the former will overestimate age-specific mortality rates for the dispersing sex, and doing the latter will greatly underestimate age-specific mortality rates because of heavy censoring. These two time-linked processes (death and dispersal) are a form of competing risks^13^. For accurate mortality estimates, we recommend (and are in the process of developing) methods of treating these ‘event-linked’ disappearances in a probabilistic manner.

## Usage Notes

**Figure 3:**
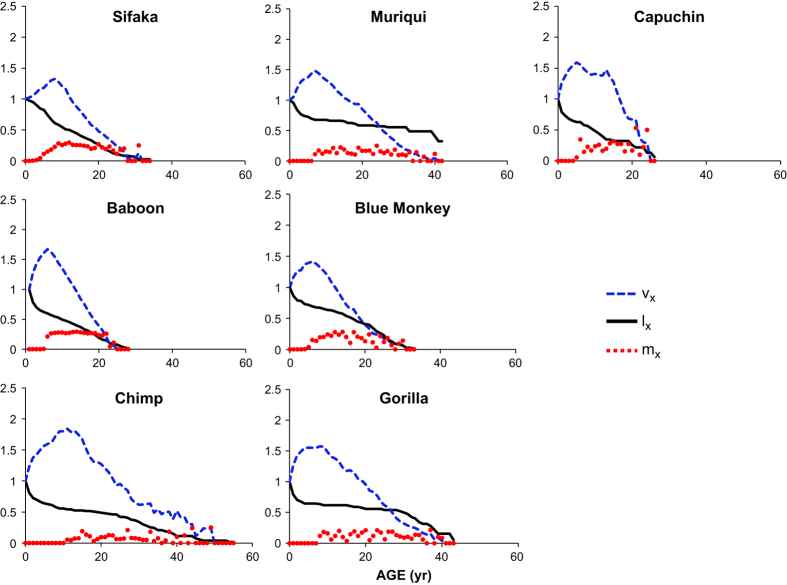
Age-specific survival (*l*_*x*_), fertility (*m*_*x*_), and reproductive values (*v*_*x*_) for seven species of primate. Reproductive value is set to 1 at birth, increases steadily until age of first reproduction, and then declines steadily until the end of life for all species.

**Figure 4:**
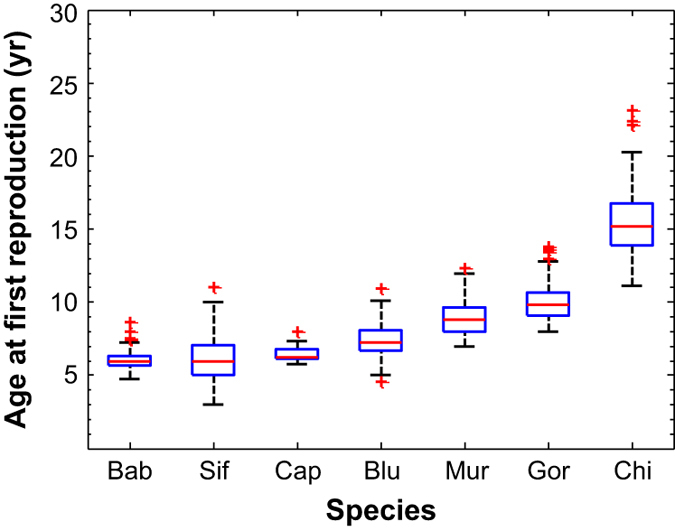
Distribution of female age (yr) of first reproduction. In this box-and-whiskers plot, red lines show the median, blue boxes show the interquartile range, whiskers extend to the most extreme data point which is ≤1.5 times the interquartile range from the box, and red plus symbols indicate more extreme data points. Species are ordered from lowest to highest age at first reproduction. Age was estimated for females present at the onset of a study and for those who immigrated into a study group, which may have introduced error into these estimates of age at first reproduction.

**Figure 5:**
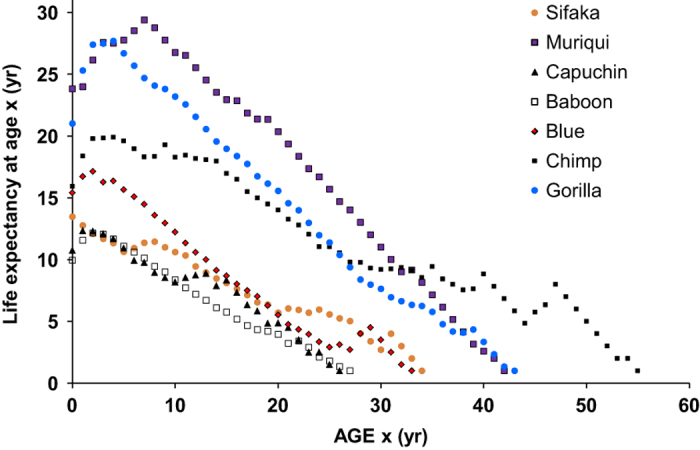
Female Life expectancy at age *x* (*e*_*x*_) versus *x* (in years).

These life tables will be useful in the fields of comparative biodemography, life history evolution, and conservation biology. For example, the field of life history evolution seeks to understand how variation in the trajectories of mortality and fertility influence: reproductive values or the extent to which females have a post-reproductive lifespan (Fig. 3); variation in age of first reproduction (Fig. 4); and variation in life expectancy with age (Fig. 5). Another question these data can help to answer is how generation time and population growth rates are correlated in a phylogenetic context across different lineages of amniotes^14,15^. As was the case for mortality estimates^3^, the population parameters we have calculated show no relationship to the phylogeny of these seven species. Nor do these population parameters correlate with body mass. Life tables (or their Leslie matrix equivalents, see Table 4 (Data Citation 1) for an example of converting a life table to a Leslie matrix for female baboons) provide invaluable tools for designing strategies to manage populations of threatened and endangered species^10,16^. (To convert the life table to a Leslie matrix, place the m_x_ values in the first row of a matrix, and calculate l_(x+1)_/l_x_ and place each value on the subdiagonal in column x. Place zeroes everywhere else.) Many wild primate populations are currently threatened; we hope that the life tables presented here will assist in conserving these charismatic species.

Finally, we note that we have published a number of different papers reporting age-specific mortality rates for these species^3,4^. Not all of these reports will agree with each other, or with the data presented here, because our database is continually growing, which results in improved estimates on a regular basis.

## Additional Information

**How to cite this article:** Bronikowski, A.M. *et al.* Female and male life tables for seven wild primate species. *Sci. Data* 3:160006 doi: 10.1038/sdata.2016.6 (2016).

## Supplementary Material



## Figures and Tables

**Table 1:** Study species information.

**Common name (3 letter code used in figures)**	**Species**	**Family**	**Country**	**Study Start Year**	*Study End Date	**Sample Size**		**Age at onset of Adulthood (years)**		**Predominant dispersing sex**	**Avg age of first dispersal (yrs)**	**Avg adult** † **body mass F, M (kg)**
						**M**	**F**	**M**	**F**			
Sifaka (Sif)	*Propithecus verreauxi*	Indriidae	Madagascar	1984	8/13/2013	342	266	5–6	6–7	M	4–5	2.76, 2.84
Muriqui (Mur)	*Brachyteles hypoxanthus*	Atelidae	Brazil	1983	9/30/2013	263	263	6–7	8–9	F	6–7	8.33, 9.42
Capuchin (Cap)	*Cebus capucinus*	Cebidae	Costa Rica	1983	8/31/2013	158	113	6–7	6–7	M	4–5	2.28, 3.23
Yellow baboon (Bab)	*Papio cynocephalus*	Cercopithecidae	Kenya	1971	6/28/2013	706	618	7–8	5–6	M	7–8	12.8, 24.4
Blue monkey (Blu)	*Cercopithecus mitis*	Cercopithecidae	Kenya	1979	8/31/2013	204	255	8–9	7–8	M	7–8	4.2, 6.8
Chimpanzee (Chi)	*Pan troglodytes*	Hominidae	Tanzania	1963	10/31/2013	133	155	14–15	14–15	F	12–13	31.3, 39
Gorilla (Gor)	*Gorilla beringei*	Hominidae	Rwanda	1967	9/30/2013	152	150	15–16	9–10	M	15–16	95, 160
										F	7–8	

*Data are continuous through this date.

^†^ Data as summarized in ref. 3.
